# *Pseudomonas* spp. in Canine Otitis Externa

**DOI:** 10.3390/microorganisms11112650

**Published:** 2023-10-28

**Authors:** Bailey Secker, Stephen Shaw, Robert J. Atterbury

**Affiliations:** 1 School of Veterinary Medicine and Science, University of Nottingham, College Road, Sutton Bonington, Leicestershire LE12 5RD, UK; bailey.secker@nottingham.ac.uk (B.S.); steve.shaw@nottingham.ac.uk (S.S.); 2 School of Biosciences, University of Nottingham, College Road, Sutton Bonington, Leicestershire LE12 5RD, UK

**Keywords:** *Pseudomonas*, canine, otitis externa, dog, companion animal

## Abstract

Canine otitis externa (OE) is a commonly diagnosed condition seen in veterinary practice worldwide. In this review, we discuss the mechanisms of the disease, with a particular focus on the biological characteristics of *Pseudomonas aeruginosa* and the impact that antibiotic resistance has on successful recovery from OE. We also consider potential alternatives to antimicrobial chemotherapy for the treatment of recalcitrant infections. *P. aeruginosa* is not a typical constituent of the canine ear microbiota, but is frequently isolated from cases of chronic OE, and the nature of this pathogen often makes treatment difficult. Biofilm formation is identified in 40–95% of *P. aeruginosa* from cases of OE and intrinsic and acquired antibiotic resistance, especially resistance to clinically important antibiotics, highlights the need for alternative treatments. The role of other virulence factors in OE remains relatively unexplored and further work is needed. The studies described in this work highlight several potential alternative treatments, including the use of bacteriophages. This review provides a summary of the aetiology of OE with particular reference to the dysbiosis that leads to colonisation by *P. aeruginosa* and highlights the need for novel treatments for the future management of *P. aeruginosa* otitis.

## 1. Introduction

### 1.1. Canine Otitis Externa

Otitis externa (OE) is defined as inflammation of the external ear canal that may affect the entire length, or any part, of the canal from the tympanic membrane (TM) to the outer meatus, and is often associated with concurrent pinnal changes. The disease can be painful and/or pruritic, acute, or chronic, and may affect one or both ears. Chronic OE is defined variably, but is usually considered to be disease of over three months duration [1], or which returns within three months and/or for the ear to demonstrate changes consistent with chronic disease [2].

OE is a very common condition in dogs presenting to veterinary clinics worldwide [3]. Secondary infections, particularly those involving *Pseudomonas* spp., most commonly *Pseudomonas aeruginosa*, are key in disease progression, morbidity, and treatment failure. Tissue changes, such as swelling and glandular hyperplasia, may result from the primary cause or secondary infection and further complicate the disease [4]. In severe cases, medical management may fail, requiring surgical intervention and resulting in hearing loss and risk of continued infection [5].

### 1.2. Normal Ear Structure and Function

The long cylindrical nature of the ear canal requires a delicate balance of the epidermal microbiota and a specialised function to maintain health [6]. Epidermal cells of the drum and ear wall are constantly being renewed. Corneocytes, shed from the surface, mix with sebaceous and ceruminous secretions to form cerumen (ear wax) that acts as a vehicle to carry material, including microorganisms, out of the ear. The underlying epidermis moves centrifugally towards the outer meatus to facilitate this [7].

A higher density of ceruminous glands has been associated with the development of OE in dogs predisposed to otitis [8,9]. These secretions largely determine the acidity of the canine ear—at around pH 6—providing a buffered environment that regulates pH changes [10]. Acute ear infections can be accompanied by a reduction in the pH of the canal wall, whereas chronic disease may be more associated with an alkaline shift. Acidity may act to control infection [11], and stronger acids—particularly acetic acid—can be used as an otic disinfectant in people as well as dogs [12,13].

The microbiota of the healthy canine ear is maintained through a complex combination of mechanical, chemical, immunological, and microbial interactions, similar to those described for the human ear [14]. Innate and specific immune responses mediated by host defence peptides, such as β-defensin (cBD3)-like and cathelicidin (cCath), and the secretion of IgA, IgM, and (particularly) IgG in cerumen contribute to this [15]. Bacterial and epidermal antimicrobial peptides may act to limit bacterial growth and alter the immunosurveillance of commensal organisms. The disruption of the microbiota may lead to sustained dysbiosis and subsequent secondary infections. A further understanding of this may assist in the development of more effective treatments for OE.

### 1.3. Normal Ear Microbiota

**Figure 1:**
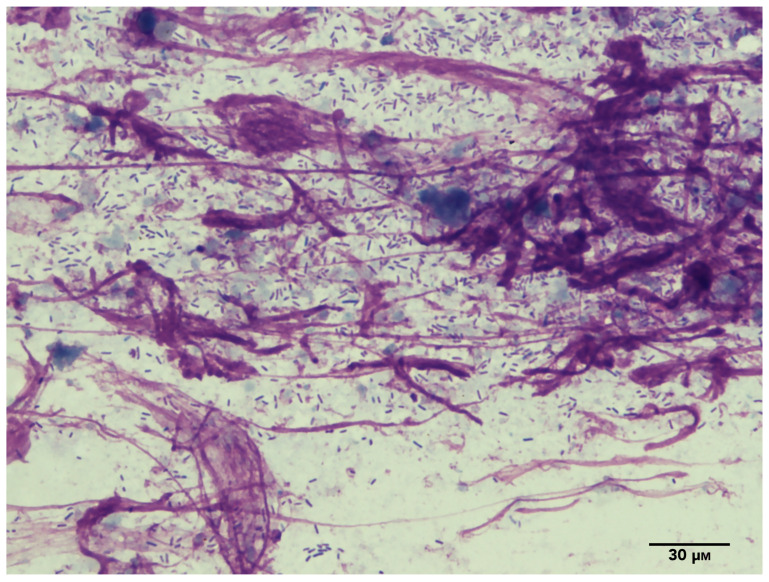
Cytology showing rods and nuclear streaming indicative of an active infection with *Pseudomonas aeruginosa*—modified Romanovsky stain (×1000).

Microorganisms in the ear are characterised using three main techniques: cytological examination, bacterial culture, and deep sequencing [16,17,18]. The cytological examination of otic discharge/cerumen is mostly used in the clinical setting as a quick, convenient test. However, this test is not sensitive and may fail to identify low or normal numbers of bacteria and yeasts [19], resulting in a binary interpretation of either normality on the one hand or infection and inflammation on the other; for instance, in *P. aeruginosa* infection, short rods are seen (Figure 1). Bacterial culture is also used in clinics, with or without the benefit of cytology, and will often identify bacteria and the non-lipid–dependent yeast *Malassezia pachydermatis*, which will grow on non-selective agar plates, regardless of whether they are in excess. When commensals are cultured, the clinical context, preferably also supported by cytology, is key to the interpretation of the laboratory results.

The organisms present in the normal canine ear have been investigated in only a few studies [16,20,21,22]. Traditional culturing has revealed the predominance of *Staphylococcus pseudintermedius*, *Bacillus* spp., coagulase-negative *Staphylococcus* spp., *Micrococcus* spp., as well as *Malassezia* spp., with smaller numbers of *Corynebacterium* spp. and Gram-negative rods [19,23,24]. Recently the microbiota of the middle ears was assessed in six healthy Beagle dogs and found to be similar to those of the external ear canal [25]. In OE, these organisms are over-represented, but the results of culture often reveal less commonly isolated organisms, such as *Staphylococcus schleiferi*, that are likely part of the normal microbiota [26]. However, as the disease progresses, transient organisms not adapted to the epidermis can invade and become established.

More recently, deep sequencing and metagenomics have revealed a richer population of bacteria and yeasts that characterise the microbiome of normal ears. They also provide evidence of changes in the microbiota that precede and follow the invasion of environmental organisms, such as *P. aeruginosa*. A metagenomic study of 257 ear swabs from 89 different dog breeds in the US (*n* = 256) and UK (*n* = 1) found *Staphylococcus pseudintermedius*, *Malassezia* spp., and *Streptococcus* spp., as would be expected from cytology and traditional culture, but also reported *Cutibacterium acnes* as commonly as *Staphylococcus pseudintermedius* [27]. Separate studies of dogs in the US, Belgium, and France found that *Corynebacterium*, *Streptococcus*, and *Staphylococcus* were frequently reported, although there was considerable variation in the microbiome between individual animals [27,28,29]. The reader is referred to other texts for a more detailed discussion of the normal microbiota of the ear [27,30].

Dysbiosis of the ear may be characterised by an overgrowth of *Malassezia* or bacterial populations, but there is not a consistent pattern of change from normal to infected ears, and this may also vary according to breed [31]. Dysbiosis often makes dogs more vulnerable to ear infections with *Pseudomonas*, particularly when accompanied by other factors, such as neglect. *P. aeruginosa* is not an obligate pathogen, and studies investigating the source of these infections often highlight environmental sources—particularly water [32], although nosocomial infections in veterinary surgery are possible [33].

## 2. Clinical Framework

**Figure 2:**
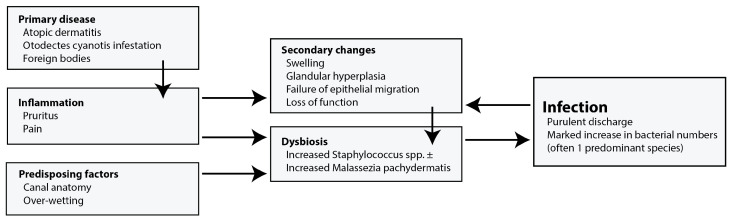
Diagram showing the influences and progress of ear disease from primary disease to infection.

August (1988) [4] devised a useful clinical framework that separated the factors involved in the development of canine OE into primary disease, predisposing factors, and perpetuating factors. This allowed a structured approach to diagnosis and treatment. More recently, the perpetuating factors have been separated into physical changes and secondary infections resulting in the PSPP (primary, secondary, perpetuating, and predisposing factors) system that is widely taught and used (Figure 2). However, this separation, though useful, is artificial and, for instance, otic inflammation and dysbiosis are intimately connected. Beyond these divisions that might be used to describe otitis, one group developed the OTIS-3 scale to describe the severity of OE using a composite score of erythema, oedema/swelling, erosion/ulceration, and exudate, but this is used infrequently [34].

### 2.1. Primary Factors

These describe the causes of initial inflammation in the ear. The most common are allergy, presence of foreign bodies, and infestations with *Otodectes cynotis*. Between 70 and 80% of dogs suffering from allergic skin disease (food and environmental allergy) have OE [35,36]. In particular, OE has been increasingly linked with canine atopic dermatitis (cAD) over the last few decades. In the 1980s, between 5 and 17% of dogs with cAD had OE [37,38], but by the early 2000s, this was reported to be up to 43% by Saridomichelakis et al. [39] and then later 50% in a large study of cAD by Favrot [40] However, this difference could be explained by a broadening of the definition of otitis to include inflammatory, as well as infected, OE [35]. It is important to note that primary factors are not identified in some cases [39].

The association between atopic dermatitis and OE complicates the PSPP system, as allergy causes inflammation and increases the probability of secondary infection. One study compared the microbiota of dogs suffering from atopic dermatitis with control animals using linear discriminant effect size analysis (LEfSe). Significantly greater numbers (*p* < 0.05) of some microbial species were found in the ears of dogs suffering from cAD compared with unaffected control animals. This was especially evident for *Staphylococcus*, which was present in 43.53% of cAD cases and 5.12% of controls. Populations of *Ralstonia*, *Methylotenera*, and *Lactobacillus* have also been reported as higher in dogs suffering from cAD [28]. Conversely, Apostolopoulos et al. [41] found lower abundances of *Brevibacterium* and *Macrococcus* (*p* ≤ 0.05) in the ear canal of German shepherd dogs with allergy, although no difference in *Staphylococcus* colonisation was reported.

The type of primary disease may influence the subsequent bacterial and yeast infections. Zur, Lifshitz, and Bdolah-Abram [42] reported *Malassezia* and rods more commonly in allergy and endocrine disease, respectively. In contrast, Paterson and Matyskiewicz [35] reported that allergy was a common cause of *Pseudomonas* infections in dogs (70%).

Despite the presence of primary disease, many dogs only present to the clinic upon the subsequent development of secondary infections with both normal skin commensals as well as environmental organisms, such as *Pseudomonas*.

### 2.2. Secondary Infections

Secondary infection usually causes a marked increase in the clinical signs experienced (increased discharge with a purulent exudate, malodour, and irritation or pain) in canine OE, and many patients are not presented until this is noted. The normal canine ear is not sterile, and depending on the primary disease, some dysbiosis may be seen in the absence of clinical infection, blurring the line between traditionally binary infected versus non-infected ears. Inflamed ears are often dysbiotic, with an overgrowth of *Staphylococcus pseudintermedius* and *Malassezia pachydermatis* [28]. In the absence of perpetuating factors (below), reducing inflammation may be sufficient to resolve the overgrowth [43].

Table 1 summarises the microbial species found in OE using culture-based and metagenomic methods. *Staphylococcus* spp. (2.97–58.5%) and *P. aeruginosa* (5.83–35.5%) are both frequently isolated using culture, along with the fungus *Malassezia pachydermatis* (8.75–30.9%). Shotgun metagenomics has further identified the obligate anaerobes *Peptostreptococcus canis* and *Porphyromonas cangingivalis* in 5.52% and 4.38% of OE cases respectively [27]. The anaerobic nature of these organisms means that they have not yet been implicated in cases of OE; however, they have both been identified in the canine mouth [44,45].

*Corynebacterium* spp. Was isolated from 0.79% of canine OE patients in Bulgaria [46]. However, metagenomic sequencing suggests that this could be an underestimate, as its prevalence could be up to 5.4–7.08% [27,29]. This is in line with other studies showing the greater sensitivity of 16S rRNA sequencing compared with traditional culture methods [18]. However, the results of DNA sequence analysis should be interpreted with caution, as they do not distinguish between viable and dead cells.

**Figure 3:**
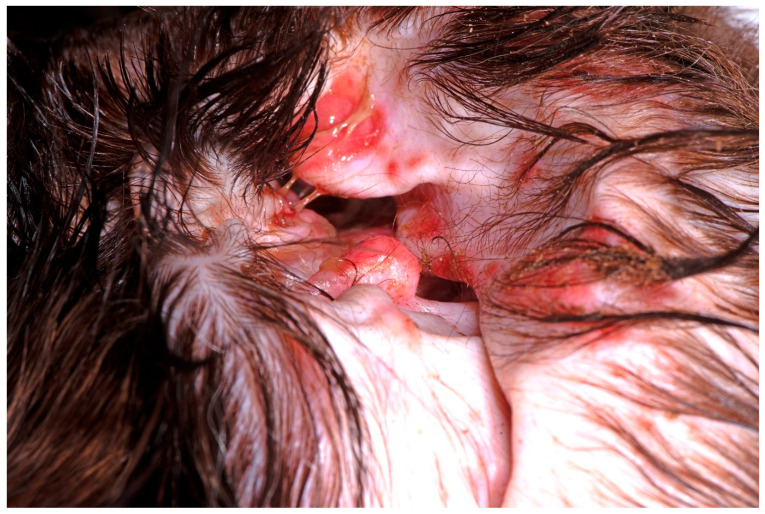
An example of the clinical appearance of *Pseudomonas* otitis in a Cocker spaniel.

Given the microbial diversity of healthy ears and the range of environmental and physical exposures that can initiate infection, it is unsurprising that the relative abundance of pathogens in canine OE also varies considerably between studies. However, despite this diversity, *Pseudomonas* spp. are the most common bacteria in chronic otitis, with up to 35% of cases affected (Figure 3) [48]. Typically, the time from the onset of primary disease to the reporting of *Pseudomonas* infection is between 10 and 28 months [35].

### 2.3. Perpetuating Factors

Perpetuating factors represent the tissue changes in the ear canal that develop as a result of disease and that prevent the resolution of OE and make repeated infection more likely [4]. Swelling and hyperplasia of the canal wall are followed by the enlargement of the ceruminous glands (modified sweat glands) and, finally, hidradenitis. Once the disease has progressed to this stage, it is often irreversible, and with increased surface area and ineffective physical and mechanical defences, bacteria such as *Pseudomonas* thrive. Bacterial toxins and physical accumulation of discharge can cause the rupture of the tympanic membrane, allowing infection to spread to the middle ear, resulting in otitis media (OM), which can then act as a perpetuating reservoir of infection [49]. Ear canal stenosis (38%) and OM (25%) were identified as the most prevalent perpetuating factors in Greece among 100 dogs of varying breeds [39].

### 2.4. Predisposing Factors

Predisposing factors are not causative of inflammation, but increase the probability of developing otitis. Addressing these rarely results in the complete alleviation or resolution of OE once it has developed. Predisposing factors include the physical traits of the dogs such as long, pendulous, hairy, or V-shaped drop pinna, as well as narrow external canals. These traits may lead to higher levels of moisture within the ear canal, which favour bacterial growth/survival. External factors, such as swimming and over-treatment with aqueous ear cleaners, may similarly disrupt normal functioning by increasing moisture within the ear canal, disrupting the normal microbiota and predisposing to OE [39,50].

### 2.5. Prevalence

Estimates of canine OE prevalence range between 5–20% [4]. In the UK, canine OE was a frequent diagnosis (7.3%) in a random sample of 22,000 dogs attending primary care practices over a one-year period in 2016, which was second only to periodontal disease (12.52%) [51]. Further investigation revealed that certain breeds were predisposed to this disease, namely Basset Hounds, Chinese Shar Pei, and Labradoodles, whereas Chihuahuas were the least susceptible [50]. An earlier study in South-Eastern England found that OE was the most prevalent diagnosis for dogs (10.2%), followed by periodontal disease (9.3%) [52]. A similar prevalence has been reported in South Korea (6.3%) [53], the US (13%) [54], and New Zealand (7.5%) [55].

### 2.6. Treatment

Treatment for OE has three aims: reduce clinical signs, restore normal numbers and distribution of microbial organisms, and restore normal ear function. The PSPP system directs the clinician to consider the primary cause. In cases where there are minimal perpetuating changes and mild dysbiosis, removing the primary disease may be sufficient to restore ear health. Steroids are often included in the treatment of canine OE; these aid in the management of inflammation of the ear canal and are administered orally, parenterally, or topically. Although steroids do not have a direct effect on the ear microbiota [56], their use in infections, including *Pseudomonas*, is controversial as they may reduce useful inflammation and immune responses; however, severe swelling is often a marked impediment to successful treatment and they are widely used. Negative effects are offset as steroids are usually part of combined steroid, antibiotic, antifungal products (cSAA products)—see Table 2 below.

Topical antimicrobial therapy is generally preferred by clinicians for treating canine OE. This is because otic preparations provide a much higher local drug concentration than systemic treatments [57]. The concentration used is such that culture and susceptibility data must be considered carefully as the antibiotic is being used at many times the MIC. For this reason, and following cytological examination, antibiotics are often used empirically [48]. Bacterial resistance is a common feature of recurrent OE and constitutive resistance is a feature of *Pseudomonas* infections. For cases of *P. aeruginosa* OE, three classes of antibiotics are often used. These are fluoroquinolones, such as marbofloxacin, and aminoglycosides, particularly gentamicin and polymyxin B. Topical otic treatments containing antibiotics with marketing authorisation in the UK are shown in Table 2. In addition, some clinicians prepare ad hoc solutions using injectable solutions of antibiotics when resistance is seen or suspected [58] e.g., ticarcillin–clavulanic acid [59], amikacin [60], ceftazidime [61], and enrofloxacin [62]. There is a perception that such mixtures may be less ototoxic in the case of a ruptured tympanic membrane, but this is not substantiated in the literature [63].

Dogs with canine OE may be prescribed ear cleaners, usually as an adjunctive to antibiotics. This aids in the removal of debris from the ear canal, which is important for the proper function of some antimicrobials, namely aminoglycosides and polymyxin B, which have reduced efficiency in the presence of pus [57,64]. The specific composition varies between products; however, they generally include cerumenolytics, surfactants, astringents, antimicrobials, and anti-inflammatories [13], as well contain ingredients that disrupt biofilms [65]. When products with antimicrobial action are used, this can have a marked effect on the success of therapy. Such disinfectant cleaners can have a faster rate of action than antibiotics, and by providing an alternative mechanism to kill bacteria, make the development of resistance by mutant selection less likely. For fluoroquinolones, the mutant prevention concentration for *Pseudomonas* is many times the minimum inhibitory concentration and, in infections where high numbers of organisms are present, additional products are essential [66,67].

The efficacy of ear cleaners in inhibiting clinical *P. aeruginosa* from canine OE varies based on the composition. Multiple studies have failed to identify key components or properties of ear cleaners, e.g., pH. However, the combination of Tris-EDTA plus 0.15% chlorhexidine has shown some effect against *P. aeruginosa* [13,68], and products containing this formulation have been widely adopted for this reason.

In some cases of chronic OE, the disease may progress to a point where medical treatment will be unsuccessful in controlling infection. In such cases, marked canal hyperplasia, secondary OM, and biofilm production in the presence of a multi-drug-resistant *Pseudomonas* infection are often evident. At this stage, surgery is necessary, including total ear canal ablation with or without bulla osteotomy. This procedure involves the removal of the infected ear canal and bulla contents, resulting in the resolution of disease in most cases [69].

### 2.7. Environmental Prevalence

*P. aeruginosa* is often described as ubiquitous in soil and aqueous environments. However, it is more frequently isolated from soil and water sources linked to human activity, such as those contaminated with oil hydrocarbons or pesticides [70]. Urban rivers have been linked to an increased prevalence of *P. aeruginosa* [70]. In an outbreak of human OE in the Netherlands involving *P. aeruginosa*, 83% of cases were linked to swimming in freshwater lakes, even though the water met quality standards [71].

### 2.8. Pseudomonas in the Veterinary Environment

The contamination of the veterinary clinical environment has been reported in the findings of surveillance studies [72,73,74] and has been linked to life-threatening infections following cardiac and orthopaedic open surgery [75,76]. In ear disease, the contamination of otic speculums is a particular risk, chiefly in practices with poor speculum availability and high case volumes where disinfection between patients is inadequate [33,77]. This is also a problem in human ear clinics [78], although this can be addressed by using disposable speculums. There is a need for further studies to examine other parts of the otoscope system, such as handles and trailing wires, as these may pose a considerable infection risk. The impact of the accidental inoculation of *Pseudomonas* may be increased by the strategic use of narrow-spectrum antibiotic treatments containing florfenicol targeting *Staphylococcus* spp., rather than broad-spectrum products.

### 2.9. In the Home

*P. aeruginosa* has been isolated from multiple sites in the home, such as surfaces, water supplies, and dishwasher rubber seals. Several studies have identified household drains as important reservoirs of *P. aeruginosa* [79,80,81]. Clonal isolates to those causing OE in dogs have been found in the oral cavity of affected dogs and other animals in the home, as well as water bowls and taps [32], although the directionality of transmission could not be established. Clonal *P. aeruginosa* isolates have been found in household taps used to fill water bowls [32]. The same study used statistical modelling to link swimming in pools and visiting dog parks to a 64% higher prevalence of *Pseudomonas* otitis [32]. This makes sense given the occurrence of *P. aeruginosa* in environmental water samples and even in commercial swimming pools [82]. Finally, grooming products in the home and professional salons have been found to harbour *P. aeruginosa* [83]. *P. aeruginosa* can also be isolated from the ears of apparently healthy dogs, suggesting an unknown history of OE [84] or contamination from the environment. This could result in the contamination of the home environment and subsequent reinfection or infection of other animals/humans in the household.

### 2.10. Isolates

The population structure of *P. aeruginosa* causing clinical infections in animals and people is largely non-clonal, i.e., the strains involved exhibit a high degree of diversity, with little or no association between groups and diseases [85]. Very few studies have examined the genomic profiles of *P. aeruginosa* strains causing canine otitis specifically. Whole-genome MLST has revealed a high diversity of *P. aeruginosa*, with 45 different sequence types (STs) identified from 80 isolates in one study [86] and 27 STs from 29 isolates in another [87]. This mirrors studies of *Pseudomonas* from human infections, which also found a largely non-clonal population structure punctuated by highly successful epidemic clones/clonal complexes [88]. There are reports of *Pseudomonas* transmission between dogs and people, especially following contact with symptomatic dogs [89]. However, the directionality of transmission is difficult to establish, as the zooanthroponotic transmission of *P. aeruginosa* has also been reported [90].

*P. aeruginosa* isolates from canine otitis have been found to exhibit an intermediate or strong ability to form biofilms compared with isolates from other sites [91], which may contribute to their increased antimicrobial resistance (AMR) [92]. Multidrug resistance appears to be more common in isolates from dogs (35%) than from other animal species, namely horses and cows (0%), particularly those isolated from cases of otitis [85]. However, stable clonal variants of *P. aeruginosa* that exhibit either enhanced biofilm formation or accelerated detachment may coexist in the same clinical sample. Greater production of extracellular polysaccharides has been found to positively correlate with the intracellular levels of the secondary messenger cyclic di-GMP in clinical *P. aeruginosa* canine otitis isolates [93]. The coexistence and cooperation of these variants may enhance bacterial persistence overall in what has been described as the “insurance hypothesis” [94].

## 3. Toxin Production/Virulence Factors

Few studies have investigated virulence factors specific to the development of canine OE. While it is widely accepted that biofilm formation and antibiotic resistance contribute to *P. aeruginosa* infection in canine OE [57], other virulence factors are relatively unexplored. In insect and plant infection models, clinical (hospital) and environmental (soil and water) isolates showed no statistically significant difference in pathogenesis [95], supporting the notion of infections originating from exposure to environmental reservoirs.

Biofilm formation is likely the most important virulence factor involved in chronic *P. aeruginosa* canine OE infections and is associated with poor treatment outcomes [92,96,97]. Biofilms provide protection from antimicrobials and the immune system, and facilitate dispersion to other environments (for a detailed review, see [98]). In cases of canine OE, this often presents as the development of OM, which can subsequently re-infect the ear canal [1]. Bacteria form biofilms by attaching to a surface and producing extracellular polymeric substances comprising exopolysaccharides, extracellular DNA, and proteins. Biofilms are produced by 40–95% of *P. aeruginosa* isolates from canine OE [92,96,97] and 92% of human patients with chronic OE, but only 20% from those with acute otitis [99]. *Pseudomonas* isolates from human OE showed increased adherence to guinea pig epithelial cell lines compared with isolates from other sites of infection [100]. The removal of the biofilm using novel therapies, such as chemical ear peeling, significantly reduced the recurrence of symptoms compared with ciprofloxacin/hydrocortisone antibiotic treatment in humans [99].

Hattab et al. [91] investigated the presence of five virulence genes in *P. aeruginosa* isolates from dogs, including from the ear canal. Three genes, *lasB* (elastase A), *aprA* (alkaline protease), and *plcH* (haemolytic phospholipase C), were present in all of the tested isolates, while *exoS* (bi-functional type-III cytotoxin) and *toxA* (Exotoxin A) were present in 87.5% and 91.7%, respectively [91]. Similarly, *P. aeruginosa* isolated from human OM patients in Egypt was positively amplified between two and ten of the quorum sensing (*rhlR*, *rhlI*, *lasR*, *lasI*) and virulence genes (*lasB*, *toxA*, *aprA*, *algD*, *exoS*, and *plcH*) [101]. While these findings may help to identify areas for further investigation, we need to determine if/when these genes are expressed to establish their role in disease. Details of *P. aeruginosa* virulence and secretion systems have been reviewed elsewhere [102] and this review will concentrate on those factors with specific relevance to canine OE.

Bacterial proteases are a major contributor to disease. *P. aeruginosa* secretes several proteases, including alkaline protease (AprA), Elastase A and B (LasA and LasB), protease IV (PrpL), small protease (PASP), large exoprotease (LepA), and others [103]. Protease activity, measured by the degradation of azocasein, from *P. aeruginosa* infection has been identified in humans with OM [104]. The total protease activity of *P. aeruginosa* isolates from canine OE is more variable than that from other animal sources (*p* < 0.0001), but is the same on average (*p* = 0.7538) when assessed by the hide powder azure absorbance assay [105]. The treatment of a chinchilla model of *P. aeruginosa* OM with bacterial protease inhibitor (GM 6001) resulted in higher survival (66%) compared with the control, gentamicin and gentamicin + GM 6001 groups, although this was not significant (*p* = 0.2674) [106].

*P. aeruginosa* isolates from chronic cases of canine OE have been found to have a reduced mean elastase activity compared with other animal isolates (*p* < 0.0001); moreover, some strains produce a stable elastase-negative phenotype due to deficiencies in the *rhl* quorum sensing system. Interestingly, the *rhl* phenotype was observed in an isolate with wild-type elastase activity, implicating an unknown constituent of quorum sensing being important in chronic otitis infections [105,107].

Haemolytic phospholipase C (*plcH*) is an exoenzyme that is able to lyse red blood cells [108] in addition to components of eukaryotic cell membranes [109]. No specific studies have been performed investigating the role of *plcH* in cases of OE. The intradermal inoculation of the protein in an in vivo mouse model resulted in a concentration-dependent response showing either no change, erythema, or dermonecrosis [110].

Bacterial secretion systems are important for the virulence of Gram-negative bacteria as they allow for the transport of proteins across the two membranes and, in some cases, directly into a target cell. Exotoxin A (ToxA) is exported via the SEC-dependent type II secretion system. The toxin is part of the AB toxin family, where the A subunit has enzymatic activity and the B subunit facilitates attachment to CD91 (also known as alpha2-macroglobulin receptor/low-density lipoprotein receptor-related protein α2MR/LRP). Once inside the host cytoplasm, the protease furin cleaves the protein and it is transported to the trans Golgi network, where it inhibits the eukaryotic elongation factor-2 by ADP-ribosylation, inducing apoptosis. This allows the toxin to bind to and kill cells from many different tissues [111,112]. Increased transcription of exotoxin A in human OE patients has been associated with patients suffering from more severe symptoms compared with those with ‘mild to moderate’ symptoms [113]. Additionally, when applied to the middle ear, the toxin causes the apoptosis of epithelial cells and can penetrate the inner ear, causing damage to the cochlea in multiple animal studies [114,115,116]. Whether the toxin is able to cross the tympanic membrane during OE and have this effect has not been investigated.

ExoS is part of the type III secretion system of *P. aeruginosa*. It possesses an N-terminal GTPase-activating protein region that triggers actin cytoskeleton disruption, and a C-terminal adenosine diphosphate ribosyl transferase, which is a major cause of host apoptosis [117]. The presence of ExoS is commonly mutually exclusive with ExoU, although the reason for this and its role in infection are unclear [118,119]. *P. aeruginosa* isolates from human chronic OM cases were significantly more likely to be *exoU*+ than *exoS*+ compared with a control group of isolates from blood and respiratory infections [120]. In human cases of OE, *P. aeruginosa* isolates have been shown to produce significantly less pyocyanin and alginate than isolates from other sites of infection, in addition to increased deoxyribonuclease production [100]. Whether differences in these factors are seen in canine OE and their clinical significance remains to be elucidated.

## 4. Antibiotic, Disinfectant, and Biological Control

In addition to biofilm formation, the outer membrane of *P. aeruginosa* has limited permeability, which confers some intrinsic resistance to antimicrobials and allows specific antibiotic resistance mechanisms to act more effectively. Resistance can also arise from efflux pumps and the expression of a chromosomal β-lactamase [121].

AMR, especially for Gram-negative rods, has been recognised as a problem in canine otitis for many years [122], with numerous studies documenting resistance profiles for *P. aeruginosa* isolates. Fluoroquinolone resistance has frequently been reported, particularly with respect to enrofloxacin (27–68%), orbifloxacin (55–82%), and marbofloxacin (33–35%). Gentamicin resistance seems to vary greatly depending on the study, with resistance reported in 3–43% of isolates [46,48,87,123,124,125]. Multidrug resistance, which is defined as resistance to at least one antibiotic from three or more classes [126], was recorded in 13–35% of isolates [87,123]. Non-susceptibility to carbapenem antibiotics has also been reported in 15–23% of *P. aeruginosa* isolates from canine otitis [86,123]. This is important as the World Health Organisation identified a critical need for new antibiotics against carbapenem-resistant *P. aeruginosa* [127]. Carbapenem resistance can arise in *P. aeruginosa* by either the production of carbapenemase, efflux pump over-expression, or reduced outer-membrane permeability [128]. Clinical canine otitis *P. aeruginosa* isolates producing metallo-β-lactamase VIM-2 have been identified in Korea [86].

Petrov and co-workers [46] monitored AMR patterns in dogs suffering from both Gram-positive and Gram-negative infections in OE between 2007–2011, and again in 2013–2017. They found that, for most antibiotics tested against *P. aeruginosa* between these time points, resistance had increased. Notably, gentamicin resistance increased from 2% to 15%, as did resistance to tobramycin (20% to 26%), amikacin (0% to 18%), lincomycin/spectinomycin (40% to 93%), and polymyxin B (0% to 50%). In contrast, enrofloxacin resistance decreased (38% to 27%). In France, the investigation of *P. aeruginosa* resistance profiles from canine OE surveillance databases found that resistance to gentamicin remained unchanged over a 5-year period, while resistance to enrofloxacin first increased and then slowly decreased over the following years. Worryingly, resistance to both enrofloxacin and gentamycin was reported in 19.4% of isolates. The decrease in resistance to enrofloxacin was likely due to the decreased exposure of companion animals to antibiotics over the study period [125].

Park and colleagues [84] investigated the antibiotic susceptibility of *P. aeruginosa* isolates from various body sites of healthy and diseased dogs. While antibiotic resistance was more prevalent in diseased samples, resistance was still present in isolates from otherwise healthy dogs. Specifically, resistance to ciprofloxacin (10.5%) was the most common, while ciprofloxacin–gentamicin–tobramycin, gentamicin, and tobramycin all showed the same level of resistance (2.6%).

### Potential Alternative Treatments

Increasing AMR, particularly among the ESKAPE pathogens, including *Pseudomonas*, has driven research into enhancing the efficacy of current therapeutic options and the development of novel alternatives. Prior to 2000, there was very little research on alternative treatments for *Pseudomonas* OE. One study investigated the use of new antibiotic, ticarcillin in treatment-resistant cases, resulting in the initial resolution of 11 out of 12 cases, although two of these later relapsed [129]. More recently, synergy and partial synergy were reported when a combination of polymyxin B/miconazole and marbofloxacin/gentamicin was tested on *P. aeruginosa* canine otitis isolates [130,131].

Ethylenediaminetetraacetic acid (EDTA), specifically Tris-EDTA, has been studied as an adjuvant in combination with antimicrobials in relation to OE for many years [132,133,134], and is now a common component of many commercially available ear cleaners [68]. When used alone, Tris-EDTA typically produces a bacteriostatic effect on *P. aeruginosa*, unless applied in excess [97]. However, it has been used to eradicate biofilms produced by clinical *P. aeruginosa* isolates [97,135] or reduce the MIC of certain antibiotics for *P. aeruginosa* present in biofilms [65].

Tris-EDTA has been found to act synergistically with amikacin (fractional inhibitory concentration, FIC = 0.1994) and neomycin (FIC = 0.1646) against *P. aeruginosa* isolates from canine OE [133]. It has also been found to complement the action of enrofloxacin [136], marbofloxacin, and gentamicin [135] when used against AMR *P. aeruginosa* from cases of OE.

*N*-acetylcysteine (NAC) is another candidate being explored as an adjuvant for use in canine OE. Similarly to Tris-EDTA, it has been shown to inhibit the growth of *P. aeruginosa*, including in canine otitis clinical isolates [97,137,138]. Synergistic interactions between NAC and enrofloxacin or gentamicin have been observed for one *P. aeruginosa* isolate, but were indifferent or antagonistic at the concentrations tested in vitro for most isolates [137]. NAC is also able to remove biofilms formed by clinical *P. aeruginosa* (20,000–80,000 µg/mL minimum biofilm eradication concentration (MBEC)) isolates; however, it is important to note that this was at potentially ototoxic concentrations (<20,000 µg/mL) [97].

Narasin and monensin were unable to inhibit clinical *P. aeruginosa* and other Gram-negative otitis pathogens at the tested concentrations when used alone, but an additive effect for narasin (but not monensin) was seen in combination with Tris-EDTA [139,140]. However, monensin was found to significantly reduce, but not eradicate, *P. aeruginosa* biofilm development [97].

A combination of enrofloxacin plus silver sulfadiazine demonstrated increased antimicrobial susceptibility, with the mean MIC decreasing from 12.97 µg/mL to 1.52/3.05 µg/mL [141]. Similarly, clinical canine OE *P. aeruginosa* isolates challenged with silver sulfadiazine alone were all found to be susceptible, with 80% of isolates having a mean MIC of <10 µg/mL. It was noted that a commercially available product should be effective in vivo, as it has a concentration much higher than the MICs seen in the study [142].

Phytochemicals have been investigated for *P. aeruginosa* inhibition. An antimicrobial effect has been reported for cinnamon oil, cinnamaldehyde [143], oregano oil, carvacrol, thyme oil, thymol [144], basil oil, rosemary oil, clary sage oil [145], and *Harungana madagascariensis* extract [146] in vitro. In all cases, *P. aeruginosa* required a higher MIC than other common otitis pathogens, specifically Gram-positive organisms, such as *S. pseudintermedius*. One group tested an EDTA combination, and a synergistic interaction was observed for cinnamon oil (FIC = 0.27) and cinnamaldehyde (FIC = 0.26) [143]. Song and colleagues [147] showed that, when applied alone, *P. aeruginosa* was resistant to manuka oil (MIC > 8% *v*/*v*). However, when combined with Tris-EDTA, the MIC was reduced to 0.5% or less. This was also true for multidrug-resistant isolates. When investigating the use of a commercially available essential oil blend in vivo for cases of acute OE, it was reported that 33.3% of dogs were “cured” and a further 20.9% showed strong improvements. Importantly, rods were not present in the cytology; therefore, more work is needed to assess its use in chronic cases of OE involving *P. aeruginosa*. Despite this, the blend did inhibit a clinical strain of *P. aeruginosa* when tested in vitro [148].

Another promising alternative treatment is the use of antimicrobial photodynamic therapy. The use of tetra-cationic porphyrins to inactivate multidrug-resistant *P. aeruginosa* isolates from canine OE and other infection sites has been demonstrated in vitro [149,150]. Antimicrobial photodynamic therapy has been successfully used in vivo for a case of OE caused by a VIM-2 Metallo-β-lactamase-producing *P. aeruginosa* that had been unresponsive to treatment with enrofloxacin [151].

The effect of cold atmospheric microwave plasma was investigated using primarily Gram-positive canine otitis isolates, but the study did include an ATCC *P. aeruginosa*. Gram-negative bacteria, including the ATCC *P. aeruginosa*, were more susceptible to cold atmospheric microwave plasma than other isolates. For example, after 10 s of exposure at a plasma intensity of 30 W, *P. aeruginosa* survival was only 22.9%, while that of *S. aureus* was 50.3%. However, this has yet to be tested on canine OE isolates [152].

The safety and capability of in vivo bacteriophage therapy have already been assessed against antibiotic-resistant *P. aeruginosa* causing chronic otitis in humans and dogs. In humans, twenty-four patients were selected, of which twelve received bacteriophage. Those who were treated with the phage reported clinical improvement and no adverse effects, with three cases seeming to be cured after a single treatment [153]. In the canine study, ten dogs suffering from antibiotic-resistant *P. aeruginosa* otitis were treated once with a cocktail of six bacteriophages and monitored. After 48 h there was a 67% reduction in the number of *P. aeruginosa*. After 18 months, three of the animals had recovered from the disease and another three had no detectable *P. aeruginosa*; again, no side effects were reported [154]. Although these results are promising, bacteriophages are often strain-specific; therefore, a cocktail of phages targeting different receptors is required. Additionally, phage resistance may be seen, although this was not investigated in the above studies. It is also potentially important to note that the chronic nature of *P. aeruginosa* OE might require multiple rounds of phage treatment, further increasing the risk of developing resistance and therefore potentially requiring multiple phage cocktails per treatment.

## 5. Conclusions

Canine OE is a common disease in veterinary practices, with the most recent study from the UK reporting a prevalence of 7.3%. Cases are often only seen after an increase in clinical signs from secondary infection. When addressing this disease, it is important to consider the primary, secondary, perpetuating, and predisposing factors. Certain breeds are predisposed to this disease due to factors such as long, pendulous, hairy, or V-shaped drop pinna, and allergy is the most common primary factor. Advances in sequencing technologies have provided a better understanding of the natural microbiota of the canine ear and how allergies can lead to dysbiosis of the ear canal. This potentially helps us to understand why allergy is such a prevalent primary factor. Despite this, *P. aeruginosa* is still commonly isolated from cases of canine OE and, due to widely reported antimicrobial resistance and biofilm formation, still poses a major issue for clinicians. Research investigating alternative treatments has seen promising results in vitro, with only a few being tested in vivo. Further work and in vivo experiments will be required in order to provide a better prognosis for the future.

## Figures and Tables

**Table 1:** Bacteria and fungi commonly isolated from cases of canine otitis externa using both culture-based and metagenomic methods.

Genus	Species	Prevalence Using Traditional Culture	Prevalence Using Metagenomics
*Staphylococcus*	*S. intermedius*, coagulase-positive Staphylococci, *S. pseudintermedius*, *S. schleiferi*, *S. schleiferi* spp. Coagulans, coagulase-negative Staphylococci	58.5% + 6.2% [24]; 36.83% + 2.97% [46]; 24.30% [47]	22% [29]; 11.25% + 8.54% [27]
*Pseudomonas*	*P. aeruginosa*	35.5% [47]; 16.24% [46]; 7.2% [24]	18.6% [29]; 5.83% [27]
*Malassezia*	*M. pachydermatis*	30.9% [24]; 30.01% [46]	8.75% [27]
*Streptococcus*	*S. canis*, β-haemolytic Streptococci, non-haemolytic Streptococci, *S. halichoeri*, *S. agalactiae*	29.9% + 4.1% [24]; 6.2% [47]; 2.97% [46]	5.42% + 3.80% + 0.83% [27]; 2.2% [29]
*Proteus*	*P*. spp., *P. mirabilis*	14.4% [24]; 6.8% [47]; 3.56% [46]	5.60% [29]; 2.29% [27]
*Escherichia*	*E. coli*	10.30% [24]; 4.2% [47]; 3.17% [46]	
*Corynebacterium*	*C. auriscanis*, *C. freneyi*, *C.* spp.	0.79% [46]	7.08% + 4.38% [27]; 5.4% [29]
*Finegoldia*	*F. magna*		5.83% [27]
*Peptostreptococcus*	*P. canis*		5.52% [27]
*Lactobacillus*			5.5% [29]
*Enterococcus*	*E. faecium*, *E. faecalis*	5.2% [24]	2.30% [29]; 1.04% [27]
Enterobacteriaceae (Unknown genus)			4.9% [29]
*Porphyrimonas*	*P. cangingivalis*		4.5% [29]; 4.38% [27]
*Arcanobacterium*	*A. canis*		4% [27]
*Peptoniphilus*	*P. harei*		2.29% [27]
*Candida*	*C.* spp.	2.38% [46]	
*Bacillus*	*B.* spp.	0.99% [46]	
14 Others			2.08–0.63% [27]

**Table 2:** Otic treatments containing antibiotics in the UK.

Steroid	Antibiotic	Antifungal
Dexamethasone (as acetate) 0.9 mg/mL	Marbofloxacin 3 mg/mL	Clotrimazole 10 mg/mL
Prednisolone 2.5 mg/mL	Diethanolamine fusidate 5.0 mg/mL Framycetin sulphate 5.0 mg/mL	Nystatin 100,000 iu/mL
Hydrocortisone aceponate 1.11 mg/mL	Gentamicin sulfate 1505 iu/mL	Miconazole nitrate 15.1 mg/mL
Betamethasone valerate 0.88 mg/mL	Gentamicin sulfate 2640 iu/mL	Clotrimazole 8.80 mg/mL
Mometasone furoate 0.9 mg/mL	Orbifloxacin 8.5 mg/mL	Posaconazole 0.9 mg/mL
Prednisolone acetate 5 mg/mL	Polymyxin B sulfate 0.5293 mg/mL	Miconazole nitrate 23 mg/mL
Mometasone furoate 2.2 mg/dose	Florfenicol 16.7 mg/dose	Terbinafine 14.9 mg/dose
Betamethasone acetate 1 mg/dose	Florfenicol 10 mg/dose	Terbinifine 10 mg/dose

Note: no products have marketing authorisation for use in the middle ear.

## Data Availability

In this study, no new data were generated or analysed. As such, data sharing is not relevant.
